# Modeling Structural Dynamics Using FE-Meshfree QUAD4 Element with Radial-Polynomial Basis Functions

**DOI:** 10.3390/ma14092288

**Published:** 2021-04-28

**Authors:** Hongming Luo, Guanhua Sun

**Affiliations:** State Key Laboratory of Geomechanics and Geotechnical Engineering, Institute of Rock and Soil Mechanics, Chinese Academy of Sciences, Wuhan 430071, China; hmluo@whrsm.ac.cn

**Keywords:** FE-Meshfree, finite element method, FE-RPIM, mesh distortion, RPIM

## Abstract

The PU (partition-of-unity) based FE-RPIM QUAD4 (4-node quadrilateral) element was proposed for statics problems. In this element, hybrid shape functions are constructed through multiplying QUAD4 shape function with radial point interpolation method (RPIM). In the present work, the FE-RPIM QUAD4 element is further applied for structural dynamics. Numerical examples regarding to free and forced vibration analyses are presented. The numerical results show that: (1) If CMM (consistent mass matrix) is employed, the FE-RPIM QUAD4 element has better performance than QUAD4 element under both regular and distorted meshes; (2) The DLMM (diagonally lumped mass matrix) can supersede the CMM in the context of the FE-RPIM QUAD4 element even for the scheme of implicit time integration.

## 1. Introduction

The FEM (finite element method) [1] has been widely adopted to model structural dynamics. The problem domain to be considered is discretized by a series of elements of simple shapes. In a two-dimensional problem domain, finite elements are either triangles or quadrilaterals [2]. TRIG3 (3-node triangular) element is much easier than QUAD4 element in mesh generation but has poorer accuracy. The QUAD4 element can generally obtain satisfactory results for regular mesh but performs badly for distorted mesh. Often in practice, the generation of a high-quality quadrilateral mesh is a time-consuming task for problems with complex geometric boundary.

A lot of work has been done in the past several decades to develop more powerful numerical methods than the FEM for the analysis of time-dependent problems. Due to the use of higher-order interpolation with specific quadrature formulae [3,4], the spectral finite element method (SFEM) is capable of simulating dynamic problems, such as wave propagation, and has better convergent rate than FEM [5]. The meshless methods, was also expected to become an effective procedure since the mesh is not needed [6,7,8,9,10,11,12,13,14,15,16]. However, some meshless methods’ shape functions have no Kronecker-delta character, which means special treatment is required for essential boundary condition implementation. Moreover, the computational cost to form the trial functions of meshless methods cannot be neglected [17]. Hence, several schemes were proposed to optimize meshless methods [18,19].

In other front, a class of PU [20] based methods were proposed, such as the PUFEM (PU FEM) [21], the GFEM (generalized FEM) [22] and the NMM (numerical manifold method) [23,24,25,26,27,28,29,30,31,32,33,34,35,36,37,38,39,40,41,42,43,44,45]. In these methods, global approximations with high order are usually built by using high order local approximations. However, the LD (linear dependence) problem [20] will arise if the local approximations and the PU functions were simultaneously constructed using explicit polynomials [21]. For the purpose of eliminating the LD problem, a lot of efforts have been made [46,47,48].

Recently, a series of FE-Meshfree elements including FE-LSPIM QUAD4 element [46], were proposed. In the FE-LSPIM QUAD4 element, shape functions are usually built through multiplying the FE shape function (QUAD4) and the meshfree shape functions (LSPIM). As a result, good properties of meshless method and FEM are inherited [46]. Compared to FEM, higher accuracy can be obtained by FE-LSPIM QUAD4 element, because global approximations with high order are constructed. In contrast to meshless method, FE-LSPIM QUAD4 shape functions have Kronecker-delta character, which means special treatment is not needed for essential boundary condition implementation. Moreover, the FE-LSPIM QUAD4 element is immune to LD problem. However, since a pure polynomial basis is adopted in the LSPIM, the moment matrix singularity may arise if the polynomial basis functions were inappropriately employed [14,49].

To avoid the drawback of LSPIM, the RPIM (radial point interpolation method) [14] has been employed to replace it, resulting in a new FE-RPIM QUAD4 element [49]. According to the report from Xu and Rajendran [49], FE-RPIM QUAD4 element has higher accuracy than FE-LSPIM QUAD4 element for linear and geometry nonlinear static problems if the same number of polynomial terms are employed. Apart from FE-LSPIM QUAD4 and FE-RPIM QUAD4 elements, there are also other types of FE-Meshfree elements [50,51,52,53].

The FE-RPIM QUAD4 element is further applied for structural dynamics in the present work. After the statement of the formulations related to FE-RPIM QUAD4 element in Section 2, equations related to elastodynamic problems are presented in Section 3. Besides, the expressions for the stiffness matrix, the CMM and the DLMM are given. In Section 4, five numerical examples are investigated using the FE-RPIM QUAD4 element. At the last section, some conclusions will be presented.

## 2. Shape Functions for FE-RPIM QUAD4 Element

### 2.1. Formulation of Shape Functions

**Figure 1:**
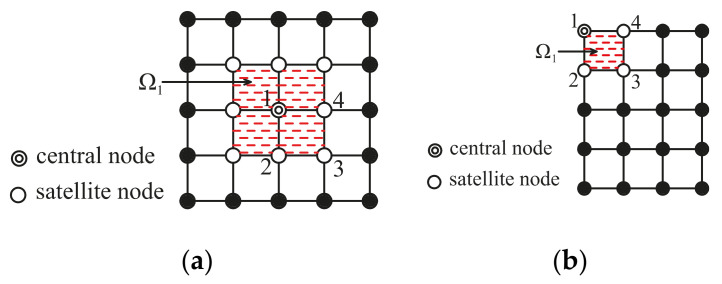
The nodal support domain of first order ($\Omega_{i}$) for a central node 1: (**a**) central node within computational domain; (**b**) central node on computational boundary.

**Figure 2:**
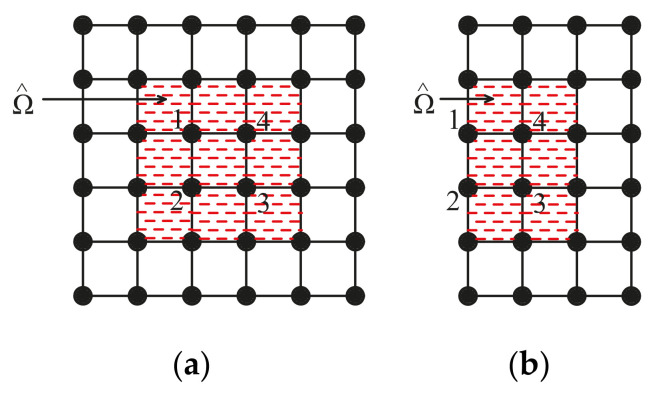
Element support domain: (**a**) central node within computational domain; (**b**) central node on computational boundary.

Before expounding formulation of the shape functions, two important concepts, namely, the nodal support domain ($\Omega_{i}$) and the element support domain (${\hat{\Omega }}^{e}$), are introduced. The nodal support domain is adopted to determine all the support nodes of a given node (also named as central node) to construct its local approximation. According to the scope of the support domain, we can define different order of nodal support domain ($\Omega_{i}$). The nodal support domain of first order is determined through the nodal connectivity of first order. Figure 1 shows an example for the nodal support domain of first order for node 1, where 9 support nodes are obtained in Figure 1a and Figure 4 support nodes are determined in Figure 1b. Similarly, the nodal support domain of second order or third order can be defined. As shown in Figure 2, $\hat{\Omega }$ is the union of 4 nodal support domains ($\Omega_{i}$), which is defined as ${\hat{\Omega }}^{e}=U_{i=1}^{4}\Omega_{i}$.

Let domain $\Omega^{e}$ be defined with nodes {*P*_1_ *P*_2_ *P*_3_ *P*_4_}. The global approximation *u^h^*(***x***) of FE-RPIM QUAD4 element can be simply written as [49]

(1) $$u^{h}\left(x\right)\;=\;w_{1}\left(x\right)u_{1}\left(x\right)\;+\;w_{2}\left(x\right)u_{2}\left(x\right)\;+\;w_{3}\left(x\right)u_{3}\left(x\right)\;+\;w_{4}\left(x\right)u_{4}\left(x\right)$$

where $w_{i}\left(x\right)$ represents PU function, while $u_{i}\left(x\right)$ represents local approximation function related to node *i*. Since quadrilateral mesh is employed, $w_{i}\left(x\right)$ is equivalent to QUAD4 shape function. It is noticed that the global approximation of QUAD4 element can be considered as a special case of FE-RPIM QUAD4 element. If $u_{i}\left(x\right)$ is set as a constant, then $u^{h}\left(x\right)$ in Equation (1) becomes the global approximation for QUAD4 element.

The local approximation functions are obtained by the RPIM [14], which is defined with:

(2) $$u_{i}\left(x\right)=\overset{M}{\underset{k=1}{\sum }}p_{k}\left(x,y\right)b_{k}+\overset{n^{\left[i\right]}}{\underset{j=1}{\sum }}r_{j}\left(x,y\right)a_{j}=pb+ra$$

where *M* and *n*^[*i*]^ represent the specified polynomial term number and the number of nodes within Ω*_i_*. **a** and **b** are two unknown vectors. **r** represents the radial basis functions, while ***p*** represents the polynomial functions formulated as:

***p***(***x***) = {1   *x   y*} for *M* = 3; (3)

***p***(***x***) = {1   *x   y   xy*} for *M* = 4; (4)

***p***(***x***) = {1   *x   y   xy   x*^2^   *y*^2^} for *M* = 6. (5)

According to the discussion in [49], the FE-RPIM QUAD4 element with three- or four-term basis can give as accurate results as with six-term basis for static problems. Furthermore, the computational cost of six-term basis or four-term basis is more than that of three-term basis. Hence, in the present paper, we set *M* = 3, namely, a three-term basis is used; **r**(*x*, *y*) can be formulated with [14,16]:

(6) $$r\left(x,y\right)=\left[r_{1}\left(x,y\right)\;\;\;r_{2}\left(x,y\right)\;\;\;\cdots \;\;\;r_{n^{\left[i\right]}}\left(x,y\right)\right]$$

in which

(7) $$r_{j}\left(x,y\right)={\left(d_{j}^{2}+c\right)}^{q}$$

in which $d_{j}\left(x,y\right)=\sqrt{{\left(x-x_{j}\right)}^{2}+{\left(y-y_{j}\right)}^{2}}$. The numerical values of *q* and *c* have an influence on the accuracy of RPIM, which have been discussed in great details in [49]. In the present work, *q* and *c* are separately set to 2.01 and 0.0001 according to the recommendation in [49].

Enforcing Equation (2) to pass through all the nodes in within Ω*_i_*, the following equations are obtained:

(8) $$u_{i}=Ra+Pb$$

where $u_{i}$ is a vector of corresponding nodal displacements of all the nodes in Ω*_i_*, and

(9) $$R=\left[\begin{array}{ccc} r_{1}\left(x_{1},y_{1}\right) & \cdots & r_{n^{\left[i\right]}}\left(x_{1},y_{1}\right)\\ \vdots & \ddots & \vdots \\ r_{1}\left(x_{n^{\left[i\right]}},y_{x_{n^{\left[i\right]}}}\right) & \cdots & r_{n^{\left[i\right]}}\left(x_{n^{\left[i\right]}},y_{x_{n^{\left[i\right]}}}\right) \end{array}\right]$$

(10) $$P=\left[\begin{array}{ccc} 1 & x_{1} & y_{1}\\ \vdots & \vdots & \vdots \\ 1 & x_{n^{\left[i\right]}} & y_{x_{n^{\left[i\right]}}} \end{array}\right]\;(M=3,\;\mathrm{so}\;\mathrm{three}\;\mathrm{columns})$$

Obviously, there are totally (*M* + *n*^[*i*]^) parameters in Equation (8). However, only *n*^[*i*]^ equations are available. Nevertheless, according to the work finished in [14,54], vectors of **a** and **b** can be eliminated as proposed in their work and local approximation function in Equation (2) is eventually expressed as [14]:

(11) $$u_{i}\left(x,y\right)=\Phi_{i}u_{i},\;\;i=1,\;2,\;3,\;4,$$

(12) $$\Phi_{i}=r\left(x,y\right)s_{a}+p\left(x,y\right)s_{b}$$

with

(13) $$\Phi_{i}=\left[\Phi_{1}^{i}\;\;\;\Phi_{2}^{i}\;\;\;\cdots \;\;\;\Phi_{n^{\left[i\right]}}^{i}\right]$$

(14) $$u_{i}={\left[u_{1}\;u_{2}\;\;\;\cdots \;\;\;u_{n^{\left[i\right]}}\right]}^{T}$$

(15) $$s_{b}={\left[P^{T}R^{-1}P\right]}^{-1}P^{T}R^{-1}$$

(16) $$s_{a}=R^{-1}\left[I-Ps_{b}\right]$$

(17) $$a=s_{a}u_{i}$$

(18) $$b=s_{b}u_{i}$$

Note that Wendland [55] has presented the evident of the existence of $R^{-1}$ for any scattered nodes.

### 2.2. Properties of Shape Functions

Through substituting Equation (11) into Equation (1), $u^{h}\left(x\right)$ for the FE-RPIM QUAD4 element can then be formulated with a more compact form:

(19) $$u^{h}\left(x\right)=w_{1}\left(x\right)\Phi_{1}u_{1}+w_{2}\left(x\right)\Phi_{2}u_{2}+w_{3}\left(x\right)\Phi_{3}u_{3}+w_{4}\left(x\right)\Phi_{4}u_{4}=\overset{p}{\underset{i=1}{\sum }}\phi_{i}\left(x\right)d_{i}$$

where $\phi_{i}\left(x\right)\;$ and $d_{i}$ represent a shape function and nodal displacement associated with node *i*. *p* represents node number within ${\hat{\Omega }}^{e}$.

The attractive properties of $\phi_{i}\left(x\right)$ in Equation (19) can be listed as follows [49]:

(i) Kronecker-delta character

(20) $$\phi_{i}\left(x_{j}\right)=\delta_{ij}$$

(ii) Compatibility property at the interface of elements.
(iii) High order completeness, in other words, reproducibility of all the assumed Cartesian terms (Equation (3)).

**Figure 3:**
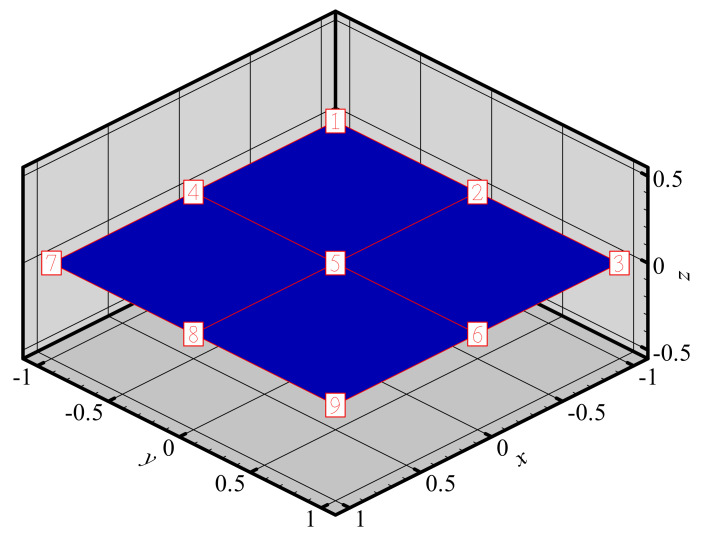
A two−dimensional domain (2 m× 2 m) discretized with 4 quadrilateral elements and 9 nodes.

**Figure 4:**
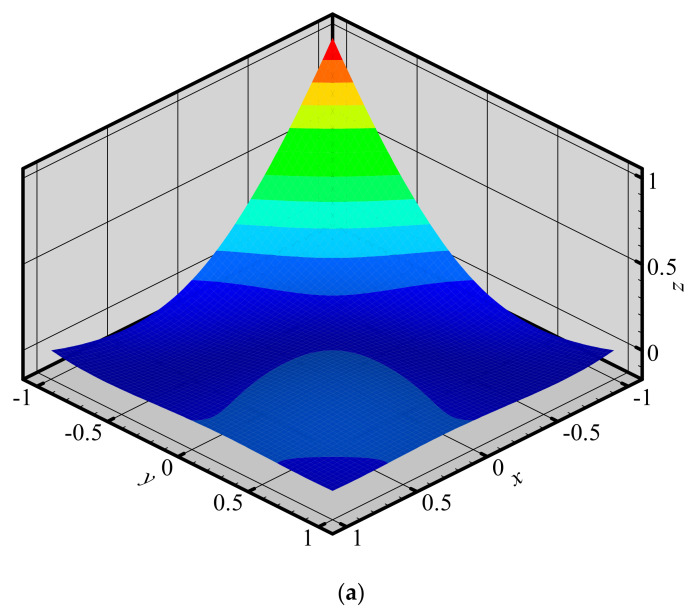

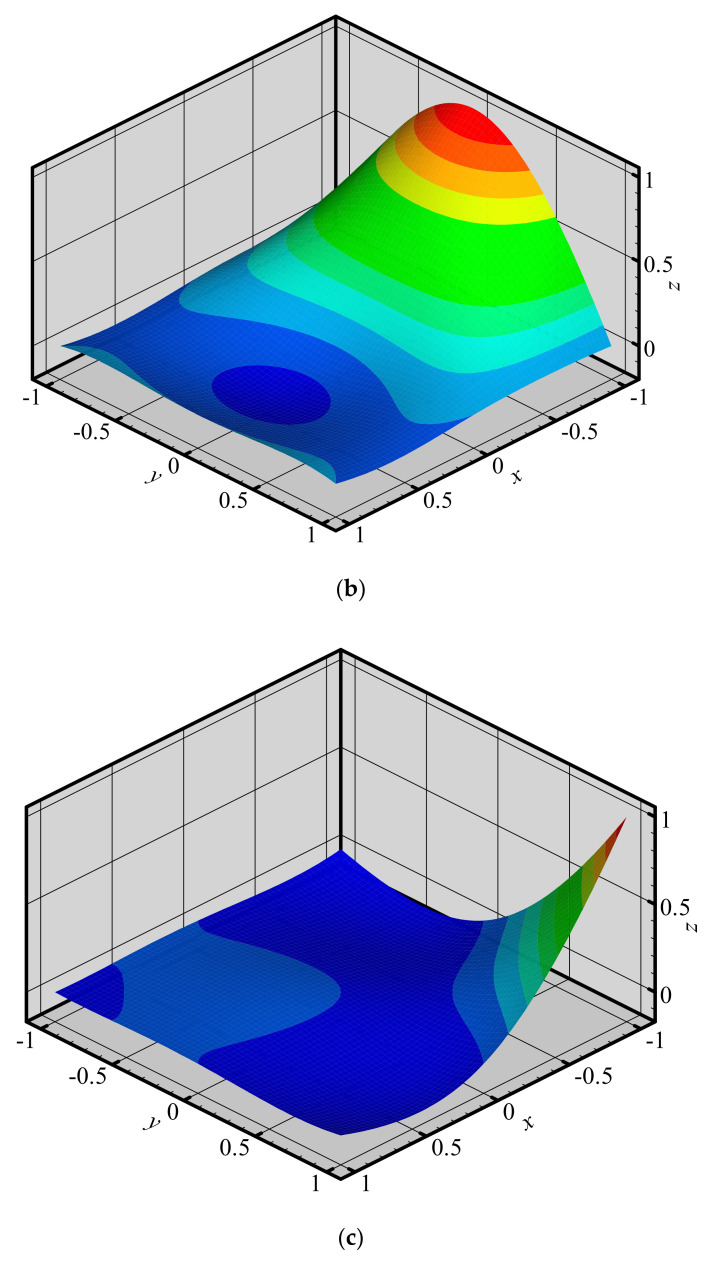

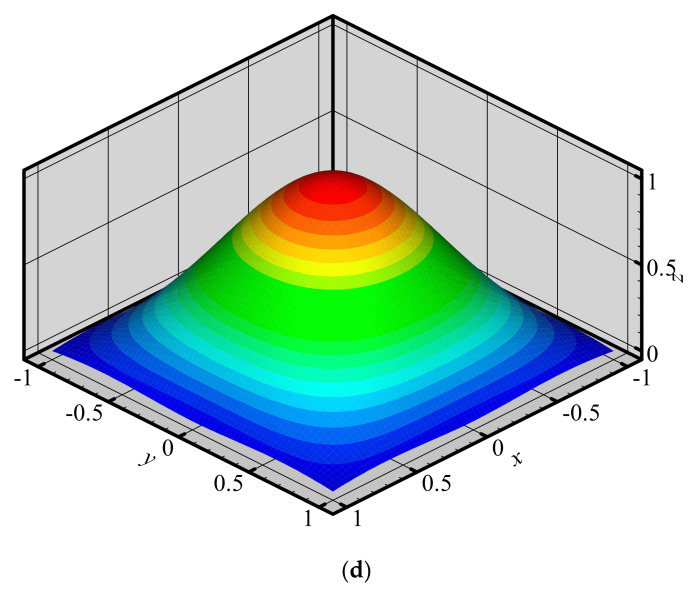
Shape functions of FE−RPIM QUAD4 element for different nodes at different locations: (**a**) node 1; (**b**) node 2; (**c**) node 3; (**d**) node 5.

**Figure 5:**
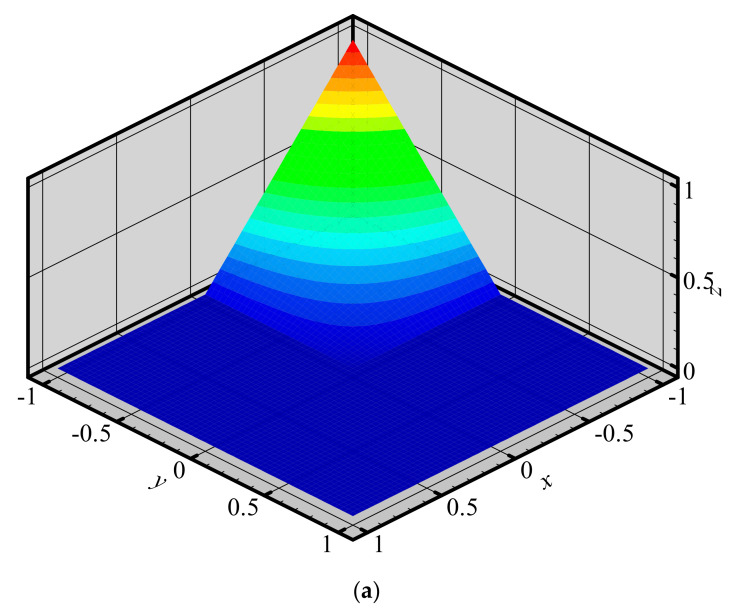

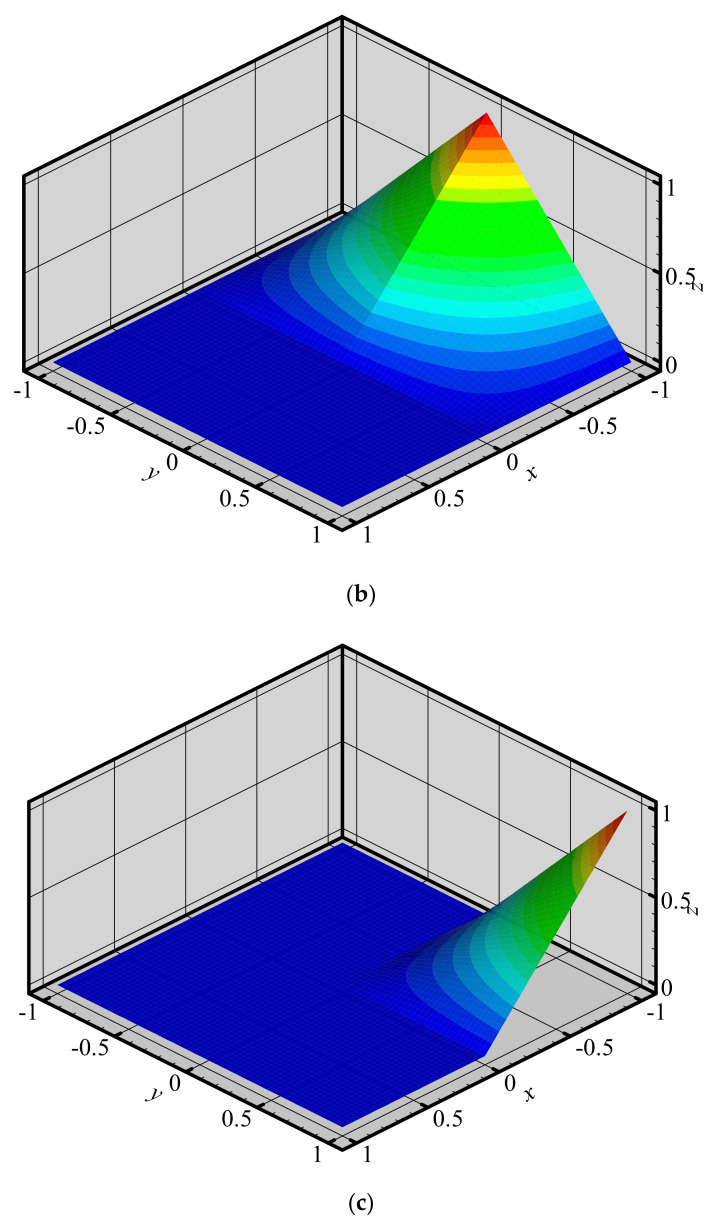

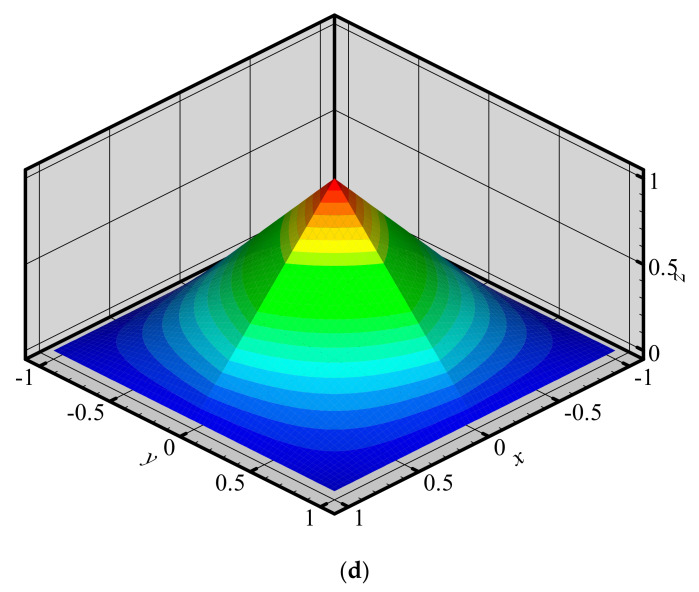
Shape functions of QUAD4 element for different nodes at different locations. (**a**) node 1; (**b**) node 2; (**c**) node 3; (**d**) node 5.

To investigate the changes of shape functions within element, we consider a two-dimensional domain discretized with four quadrilateral elements and 9 nodes (Figure 3). The 3D graphics of shape functions of FE-RPIM QUAD4 element for different nodes (node 1, node 2, node 3 and node 5) are plotted in Figure 4. For the purpose of comparison, the 3D graphics of shape functions of QUAD4 element are also plotted, as shown in Figure 5. As can be seen in Figure 4 and Figure 5, the shape function of FE-RPIM QUAD4 element is smoother than that of Quad4 element.

## 3. FE-RPIM QUAD4 for Elastodynamic Problems

### 3.1. FE-RPIM QUAD4 for Dynamic Analysis

Let the problem domain Ω discretized using a series of QUAD4 elements:$\;\Omega =\underset{e}{\sum }\Omega^{e}.$ In the context of the FE-RPIM QUAD4 element, the discretized form of the system equations about dynamic analysis can be expressed as [56]:

(21) $$M\ddot{d}+C\dot{d}+Kd=f$$

in which ***M*** is the global mass matrix, ***K*** is the global stiffness matrix, and f is the global load vector, which can be computed as:

(22) $$M=\underset{e}{\sum }M^{ce},\;\;\;\;\;\;f=\underset{e}{\sum }f^{e},\;\;\;\;\;\;K=\underset{e}{\sum }K^{e};$$

where

(23) $$K^{e}=\int_{\Omega^{e}}^{}B^{T}DBd\Omega ,$$

(24) $$M^{ce}=\int_{\Omega^{e}}^{}\rho N^{T}Nd\Omega ,$$

and

(25) $$f^{e}=\int_{\Gamma_{\sigma }^{e}}^{}N^{T}\bar{t}dS+\int_{\Omega^{e}}^{}N^{T}bd\Omega ,$$

in which ρ is the material density, ***D*** is the matrix of the elastic constants of the material, $\bar{t}$ is the specified traction vector applied on stress boundary $\Gamma_{\sigma }^{e}$, ***b*** is the body force per unit volume, N represents the matrix of shape function for $\Omega^{e}$, expressed as:

(26a) $$N=\left[\phi_{1}^{e}I,\cdots ,\phi_{p}^{e}I\right]=\left[\begin{array}{cc} \phi_{1}^{e} & 0\\ 0 & \phi_{1}^{e} \end{array},\cdots ,\begin{array}{cc} \phi_{p}^{e} & 0\\ 0 & \phi_{p}^{e} \end{array}\right],$$

and B is the gradient matrix expressed as:

(26b) $$B=\left[\begin{array}{cc} \frac{\partial \phi_{1}^{e}}{\partial x} & 0\\ 0 & \frac{\partial \phi_{1}^{e}}{\partial y}\\ \frac{\partial \phi_{1}^{e}}{\partial y} & \frac{\partial \phi_{1}^{e}}{\partial x} \end{array},\cdots ,\begin{array}{cc} \frac{\partial \phi_{p}^{e}}{\partial x} & 0\\ 0 & \frac{\partial \phi_{p}^{e}}{\partial y}\\ \frac{\partial \phi_{p}^{e}}{\partial y} & \frac{\partial \phi_{p}^{e}}{\partial x} \end{array}\right],$$

where $\phi_{i}^{e}$ represent a shape function associated with node *i*. *p* represents the node number within ${\hat{\Omega }}^{e}$.

The Rayleigh damping is adopted in the present work. Hence, the damping matrix ***C*** can be formulated into,

(27) $$C=\beta_{1}M+\beta_{2}K$$

in which $\beta_{1}$, $\beta_{2}$ represent the coefficients of Rayleigh damping.

### 3.2. Time Integration Scheme

The Newmark method [56] will be employed in this study to solve Equation (21). Assuming that $d_{n}$, $v_{n}$ and $a_{n}$ separately represent approximation values of $d\left(t_{n}\right)$, $\dot{d}\left(t_{n}\right)$ and $\ddot{d}\left(t_{n}\right)$. When $t=t_{n+1}=t_{n}+\Delta t$,

(28) $${\tilde{d}}_{n+1}=d_{n}+\Delta tv_{n}+\frac{\Delta t^{2}}{2}\left(1-2\beta \right)a_{n},\;$$

(29) $${\tilde{v}}_{n+1}=v_{n}+\Delta t\left(1-\gamma \right)a_{n},$$

then the approximations of $d\left(t_{n+1}\right)$ and $\dot{d}\left(t_{n+1}\right)$ are expressed as:

(30) $$d_{n+1}={\tilde{d}}_{n+1}+\beta \Delta t^{2}a_{n+1},$$

(31) $$v_{n+1}={\tilde{v}}_{n+1}+\gamma \Delta ta_{n+1}$$

Here, $a_{n+1}$ represents the solution of Equation (32).

(32) $$\bar{M}a_{n+1}={\bar{f}}_{n+1},$$

with

(33) $${\bar{f}}_{n+1}=f_{n+1}-C{\tilde{v}}_{n+1}-K{\tilde{d}}_{n+1},$$

(34) $$\bar{M}=M+\gamma \Delta tC+\beta \Delta t^{2}K.$$

$a_{0}$ is computed with Equation (35).

(35) $$Ma_{0}=f_{0}-Cv_{0}-Kd_{0}.\;$$

It is noticed that M obtained from Equations (22) and (24) is named as CMM (consistent mass matrix). If M is a diagonally lumped mass matrix (DLMM), solving $a_{0}$ should be a very easy task. Moreover, if the damping effect is ignored, as is done frequently and β = 0, then $\bar{M}$ reduces to M. Consequently, if M is a positive DLMM whose inverse $M^{-1}$ is easy to calculate, solving Equation (32) for $a_{n+1}$ would be very easy.

### 3.3. Generalized Eigenvalue Problem

If the terms associated to damping matrix and force vector are neglected, Equation (21) will reduce to the following form [14]:

(36) $$M\ddot{d}+Kd=0$$

The general solution for Equation (36) is formulated as:

(37) $$d=\overset{¯}{d}\exp\left(i\omega t\right)$$

in which *t* represents time. ω  represents the natural frequency, while $\bar{d}$ represents the eigenvector.

Based on Equations (36) and (37), ω is obtained from Equation (38).

(38) $$K\overset{¯}{d}=\lambda M\overset{¯}{d},\lambda =\omega^{2}$$

Here, $\bar{d}$ determines the mode shape related to  ω. Note that it is time-consuming to compute all the modes. Hence, the subspace iteration procedure is usually adopted to obtain only those modes with small values of  ω.

However, if M is a DLMM, Equation (38) becomes a standard eigenvalue problem,

(39) $$\;\tilde{K}\overset{¯}{d}=\lambda \overset{¯}{d}$$

where $\tilde{K}$ is defined as:

(40) $$\tilde{K}=M^{-1}K$$

Since M is a DLMM, the inversion of M, namely, $M^{-1}$, can be easily obtained. There are more powerful algorithms available to solve the standard eigenvalue problem (Equation (39)) [57].

### 3.4. Diagonally Lumped Mass Matrix

Here, we propose to use the “special lumping technique” introduced by Hinton et al. [58] to obtain the diagonally lumped mass matrix (DLMM). This procedure always leads to positive lumped masses at the nodes. Moreover, the requirement for mass conservation can be ensured. With little change, this procedure can be used for FE-RPIM QUAD4 element. For the FE-RPIM QUAD4 element, we obtain:

(41) $$M_{ii}^{le}=\alpha M_{ii}^{ce},\;\;\;\;\;\;i=1,2,\cdots 2p;$$

(42) $$M_{ij}^{le}=0\;\left(i\neq j\right)$$

where $M_{ii}^{ce}$ is the *i*-th diagonal entry of the element consistent mass matrix (CMM) obtained through Equation (24), $M_{ii}^{le}$ is the *i*-th diagonal entry of the DLMM, α is a constant defined as:

(43) $$\alpha =\frac{2\int_{\Omega^{e}}^{}\rho dV}{\sum_{i}M_{ii}^{ce}}$$

where ρ represents the density of material.

## 4. Numerical Examples

In this section, a static example and five examples regarding to free and forced vibration analyses will be presented to investigate the performance of FE-RPIM QUAD4 element. For comparison, other well-known element types, such as the TRIG3, QUAD4 and QUAD8 (8-node quadrilateral element) will also be used to conduct these tests. In the computation for dynamic problems, both the CMM and the DLMM will be employed in the context of FE-RPIM QUAD4 element, while only the CMM will be employed in the context of QUAD4, TRIG3, and QUAD8.

Physical units in this section will be according to the unit system of international standard without specification. *n* is the node number within discretized model. Natural frequency’s relative error expressed in Equation (44) is adopted to evaluate the accuracy of the numerical models.

(44) Re =ωnum−ωrefωref,

in which “num” and “ref” separately represent the numerical solution and reference solution.

### 4.1. Cook’s Skew Beam

**Figure 6:**
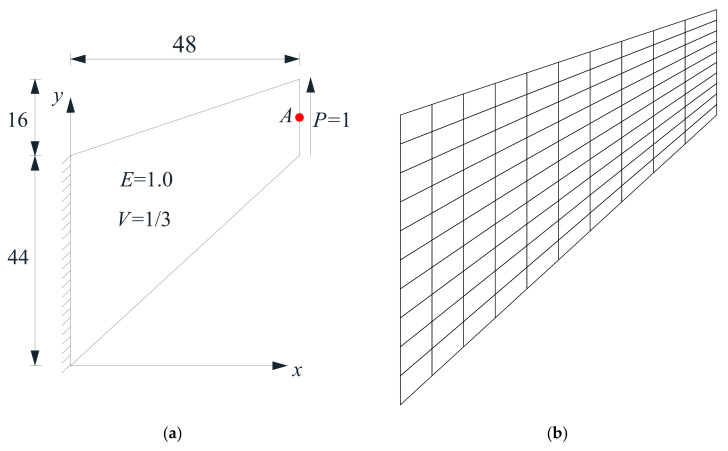
Cook’s skew beam: (**a**) geometry and load; (**b**) a mesh with 10 × 10 elements.

Before proceeding with dynamic analysis, the well-known example of Cook’s skew beam (Figure 6a) is adopted to test the performance of the FE-RPIM QUAD4 element for static problem. A mesh with 10 × 10 quadrilateral elements and 121 nodes is plotted in Figure 6b. The reference solution for the vertical displacement of point *A* for Cook’s skew beam is 23.96 [49].

With the mesh presented in Figure 6b, the solutions for the vertical displacement of point *A* based on the FE-RPIM QUAD4 element is 23.8170, which is better than that based on the QUAD4 element (22.6965). Note that this problem was also solved in [59] by the spectral finite element (SFEM) [60]. If the problem domain is discretized into 10 × 10 elements, where each element has 2 × 2 nodes, the solution based on the SFEM is 22.6940, which is inferior to that based on the FE-RPIM QUAD4 element (23.8170). However, If the problem domain is discretized into 1 element with 11 × 11 nodes, the solution based on the SFEM is 23.9414, which is even slightly better than that based on the FE-RPIM QUAD4 element (23.8170).

### 4.2. A Slender Rod

**Figure 7:**
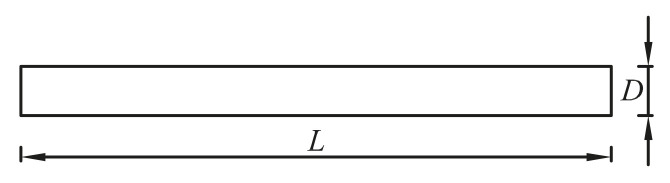
Free vibration analysis of a slender rod.

Free vibration analysis for a slender rod [61] is conducted, as shown in Figure 7. The *i*th natural frequency from the analytical solution is [62]:

(45) $$\omega =C_{0}\pi \frac{i}{L}=\sqrt{E/\rho }\pi \frac{i}{L},$$

in which *L* represents the length of the rod.

Geometric and mechanical parameters used in this example are *L* = 100, thickness *t* = 1, *D* = 1, Poisson’s ratio *v* = 0, Young’s modulus *E* = 72 × 10^9^, mass density *ρ* = 2700. Since *L*/*D =* 100 > 10, the geometric parameters used assure that the one-dimensional slender rod can be well represented by the two-dimensional model without causing unacceptable error. In the computation, both upside and down sides of the model are fixed in the normal direction.

**Figure 8:**
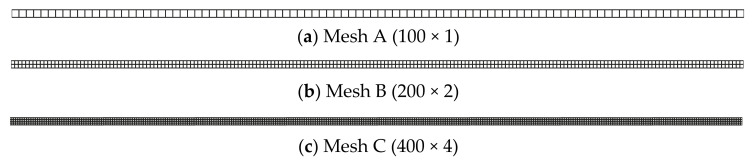
Normalized mesh for slender rod in Figure 3.

For investigating the convergence of the solution, three regular discretized models (Figure 8) are constructed. Table 1 lists the numerical results from different numerical models. According to Table 1, the results from all the listed numerical models approach the analytical solution gradually, as the mesh density increases.

By using the consistent mass scheme, the result from FE-RPIM QUAD4 element is better than that from QUAD4 element and TRIG3 element, but slightly inferior to that from QUAD8 element. Note that QUAD8 element requires more nodes than FE-RPIM QUAD4 element to discretize the problem domain. In addition, results from the CMM and the DLMM in the context of FE-RPIM QUAD4 element are close to each other.

### 4.3. An Annulus

**Figure 9:**
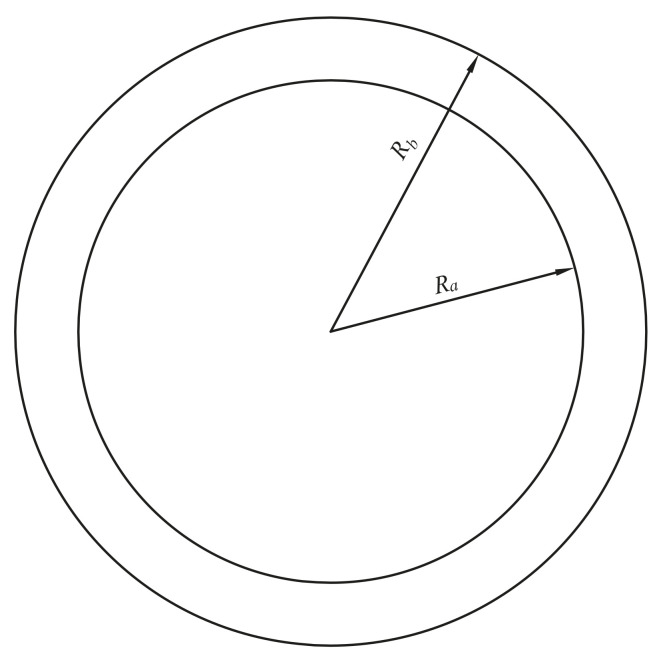
Free vibration analysis of an annulus.

As the second example, an annulus without constraint shown in Figure 9 is employed to validate the FE-RPIM QUAD4 element. The geometric and mechanical parameters used in this example are *R_a_* = 0.4, *R_b_* = 0.5, *v* = 0.33, *E* = 72 × 10^9^, *t* = 1, *ρ* = 2700.

**Figure 10:**
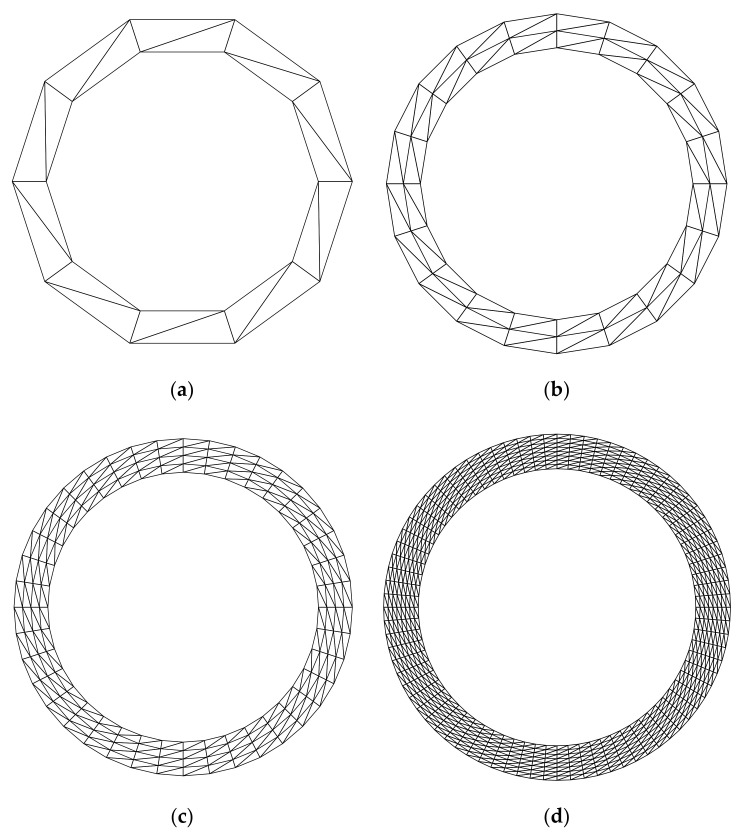
Triangular meshes for the annulus. (**a**) Mesh A (20 elements, 20 nodes); (**b**) Mesh B (80 elements, 60 nodes); (**c**) Mesh C (320 elements, 200 nodes); (**d**) Mesh D (1280 elements, 720 nodes).

**Figure 11:**
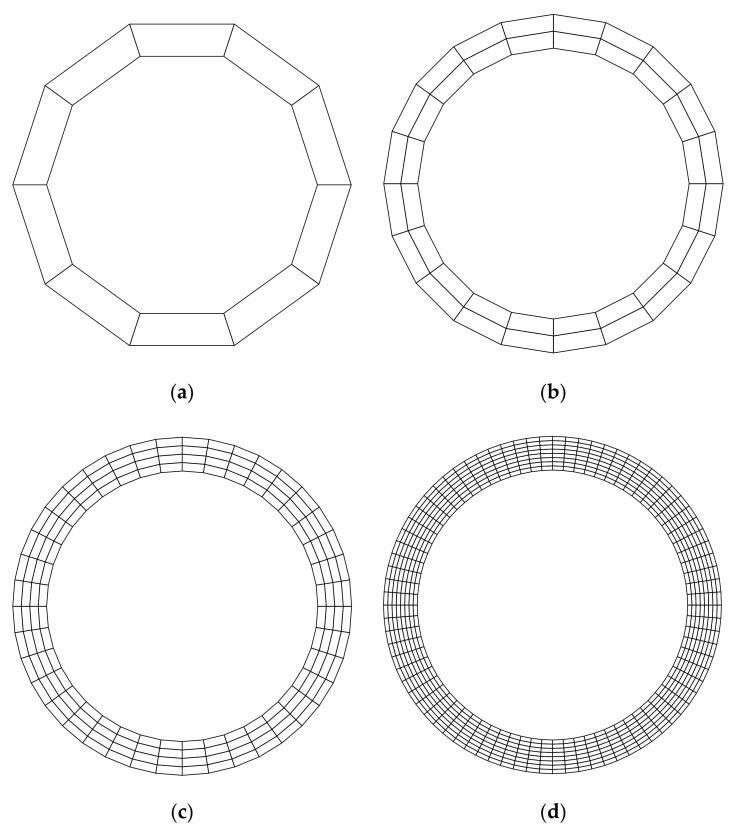
Quadrilaterial meshes for the annulus: (**a**) Mesh A (10 elements, 20 nodes); (**b**) Mesh B (40 elements, 60 nodes); (**c**) Mesh C (160 elements, 200 nodes); (**d**) Mesh D (640 elements, 720 nodes).

For the purpose of investigating the convergence of the solution, several regular discretized models shown in Figure 10 and Figure 11 are constructed. Table 2 lists the frequencies assessed by different numerical models. The reference solution for this problem, which is listed in the last column of Table 2, can be found in [61]. According to Table 2, the result from FE-RPIM QUAD4 element is much better than that from QUAD4 and TRIG3 elements, but slightly inferior to that from QUAD8 element, if the CMM was employed.

In addition, FE-RPIM QUAD4 element can obtain better result if the DLMM is employed instead of the CMM.

### 4.4. Mesh Distortion Test

**Figure 12:**
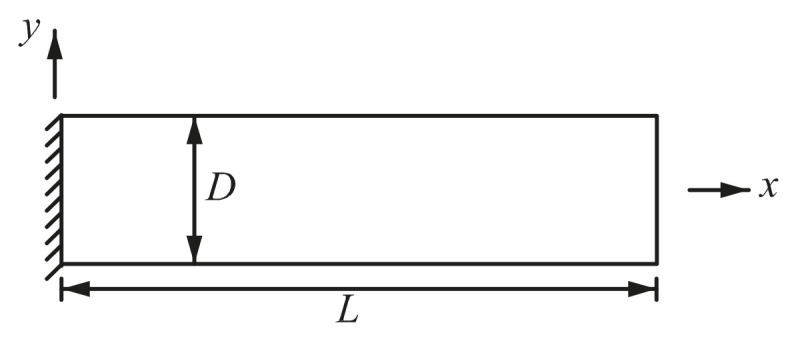
Free vibration analysis of a 2D cantilever beam.

In the previous two examples, regular meshes are adopted. In this section, mesh distortion test based on the cantilever beam (Figure 12) is conducted. The parameters used are *D* = 10 mm, *L* = 100 mm, *v* = 0.3, *E* = 2.1 × 10^4^ kg/mm^2^, ρ = 8.0 × 10^−10^ kg fs^2^/mm^4^, *t* = 1 mm.

**Figure 13:**
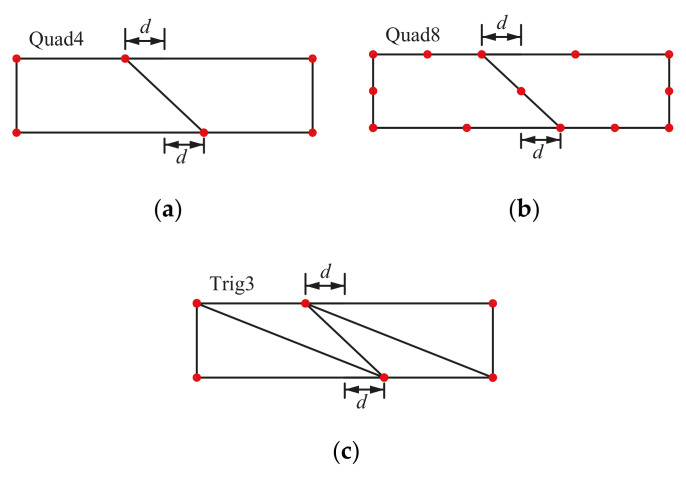
The mesh used for the distortion sensitivity test: (**a**) Mesh A; (**b**) Mesh B; (**c**) Mesh C.

As shown in Figure 13a,b, the beam is meshed with two elements for QUAD4 element, FE-RPIM QUAD4 element and QUAD8 element. For TRIG3 element, the problem domain is meshed with four elements, as shown in Figure 13c. A distortion parameter, 2*d*/*D*, is adopted to control mesh distortion.

**Figure 14:**
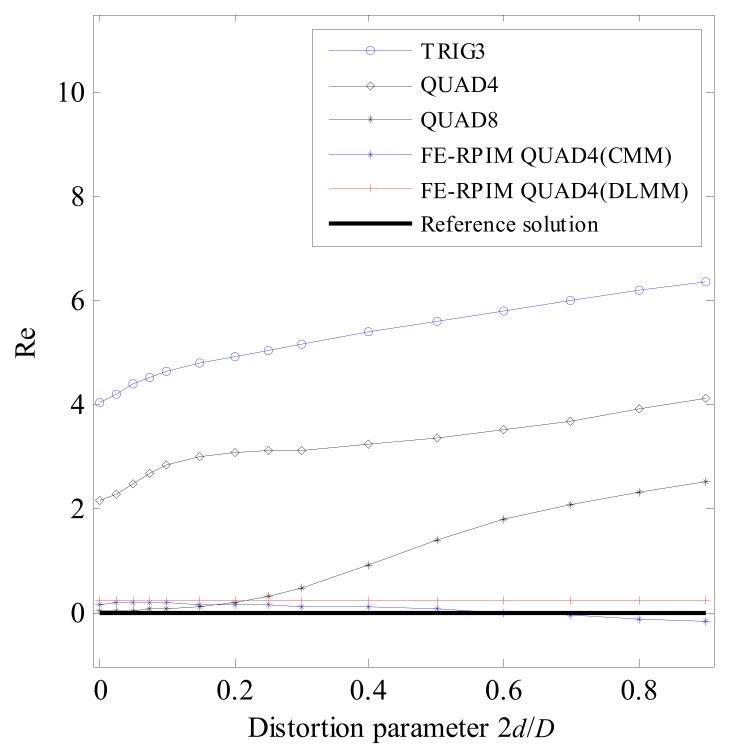
Error in fundamental natural frequency for mesh distortion sensitivity test.

**Figure 15:**
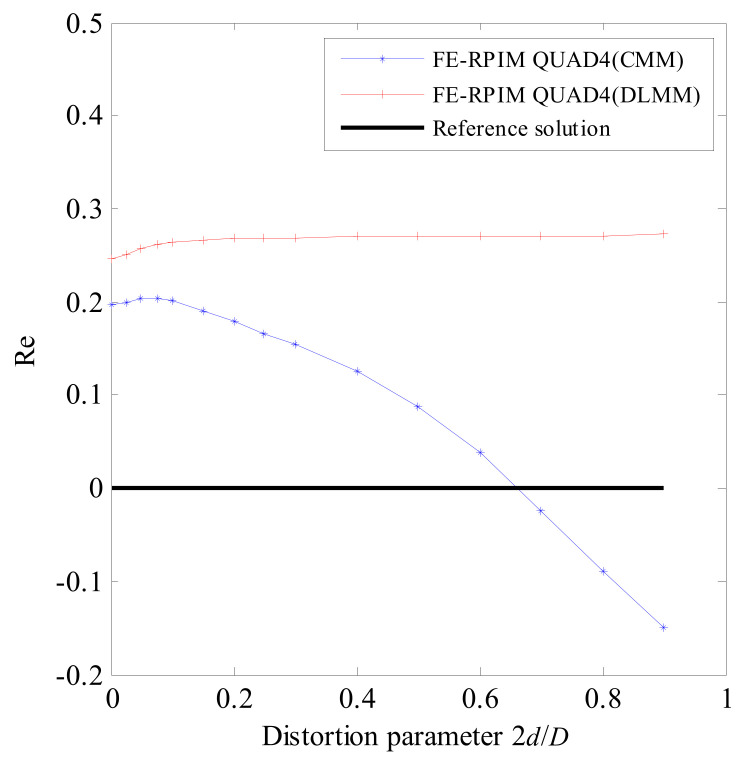
Comparison of accuracy of FE−RPIM QUAD4 (CMM) and FE−RPIM QUAD4(DLMM) for mesh distortion sensitivity test.

Table 3 lists the fundamental natural frequency assessed by different numerical models. The corresponding relative errors are plotted in Figure 14. To better see the reached accuracy of FE-RPIM QUAD4(CMM) and FE-RPIM QUAD4(DLMM), Figure 15 is plotted. According to Table 3, Figure 14 and Figure 15, we can draw the following conclusions:

(1) First, as distortion parameter’s value increases, the errors based on FE-RPIM QUAD4 element do not change appreciably, while those based on QUAD4 element, TRIG3 element and QUAD8 elements change rapidly. The FE-RPIM QUAD4 element is immune to mesh distortion.
(2) Second, accuracy of FE-RPIM QUAD4 element is always much higher than QUAD4 and TRIG3 elements.
(3) Third, when 2*d*/*D* < 0.2, QUAD8 element’s accuracy is higher than QUAD4, FE-RPIM QUAD4 and TRIG3 elements. However, as the value of 2*d*/*D* increases, accuracy through QUAD8 element deteriorates quickly. If meshes used are distorted severely, QUAD8 element’s accuracy is much lower than FE-RPIM QUAD4 element.
(4) Fourth, compared to CMM, FE-RPIM QUAD4 element can achieve better results if DLMM is employed.

### 4.5. A Plate with Four Holes

**Figure 16:**
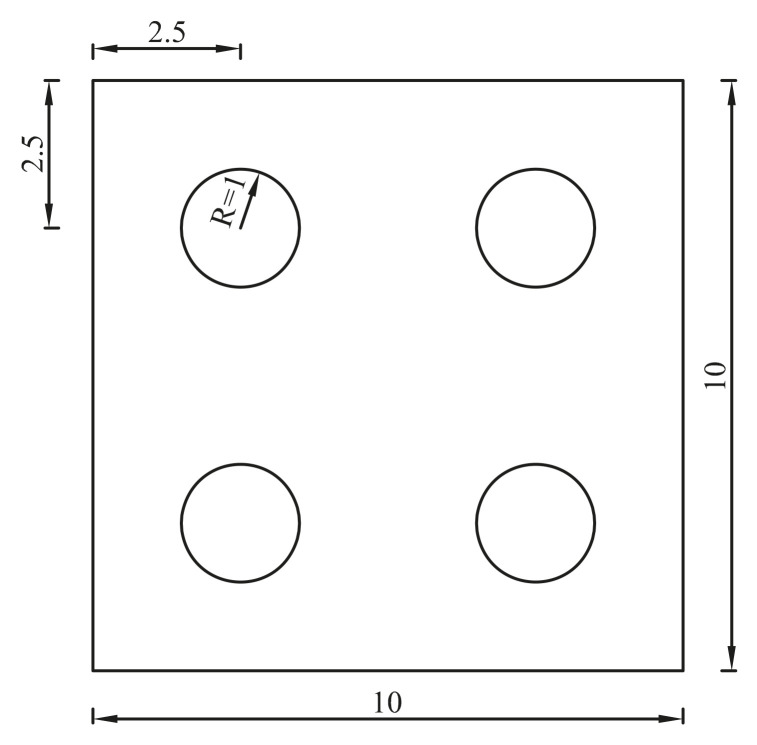
A plate with four holes.

In this section, a plate with four holes shown in Figure 16 is considered. The mechanical parameters used are *v* = 0.3, *E* = 72 × 10^9^ and *ρ* = 2700. Left side of the plate is fixed.

**Figure 17:**
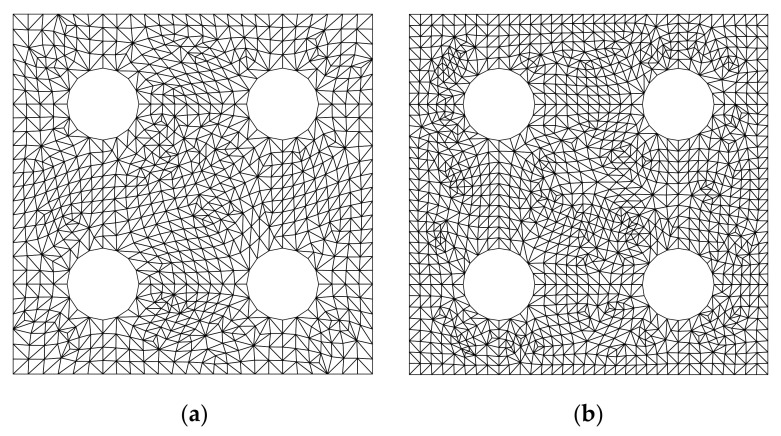

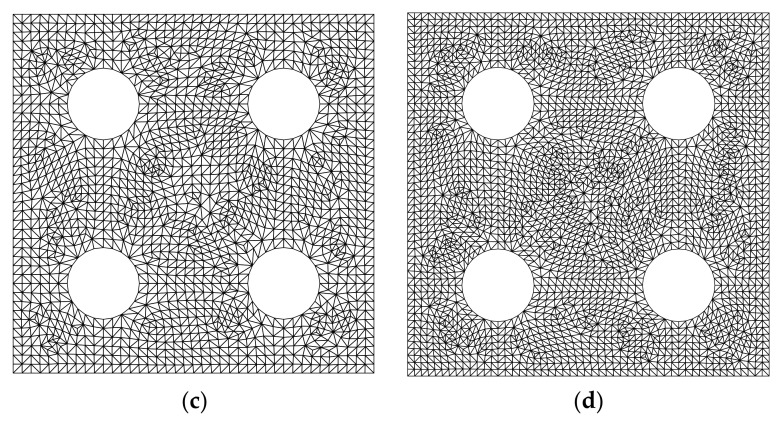
Triangular mesh for the plate with four holes. (**a**) Mesh A (1584 elements and 871 nodes); (**b**) Mesh B (2350 elements and 1278 nodes); (**c**) Mesh C (3170 elements and 1710 nodes); (**d**) Mesh D (5302 elements and 2814 nodes).

**Figure 18:**
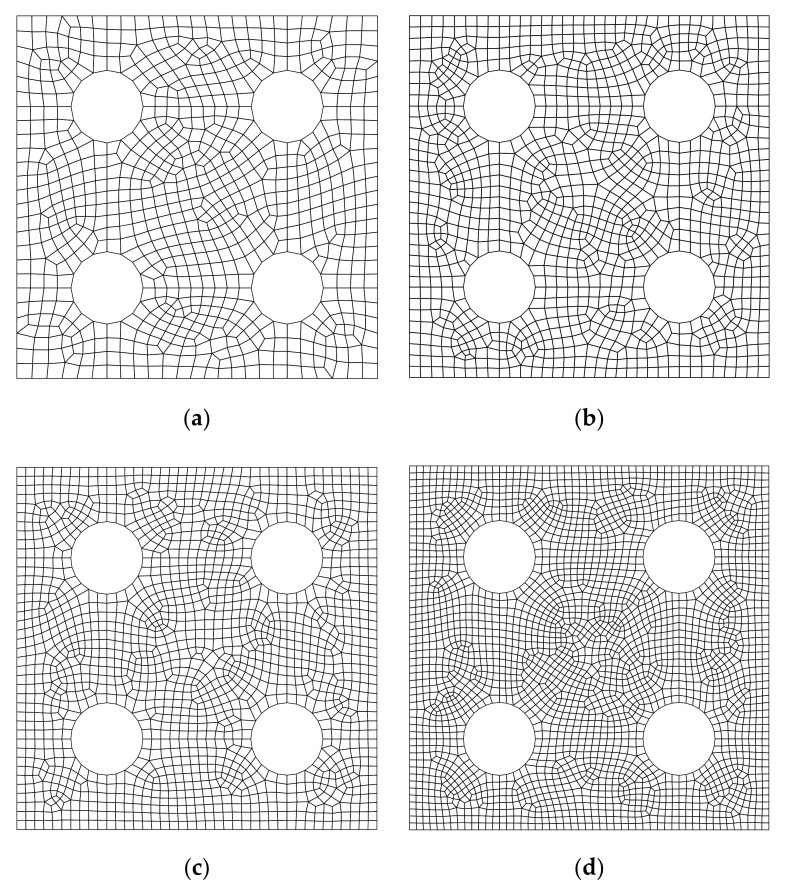
Quadrilateral mesh for the plate with four holes: (**a**) Mesh A (792 elements and 871 nodes); (**b**) Mesh B (1175 elements and 1278 nodes); (**c**) Mesh C (1585 elements and 1710 nodes); (**d**) Mesh D (2651 elements and 2814 nodes).

For the purpose of investigating the convergence of the solution, four discretized models shown in Figure 17 and Figure 18 are constructed. In order to get a reference solution, the QUAD4 element is adopted with a very dense discretized model (43,704 nodes, 43,058 elements). Table 4 lists the results from different numerical models. The results from different numerical models all approach the reference solution gradually, as the mesh density increases. In addition, by using the CMM, FE-RPIM QUAD4 element’s accuracy is much higher than QUAD4 and TRIG3 elements.

**Figure 19:**
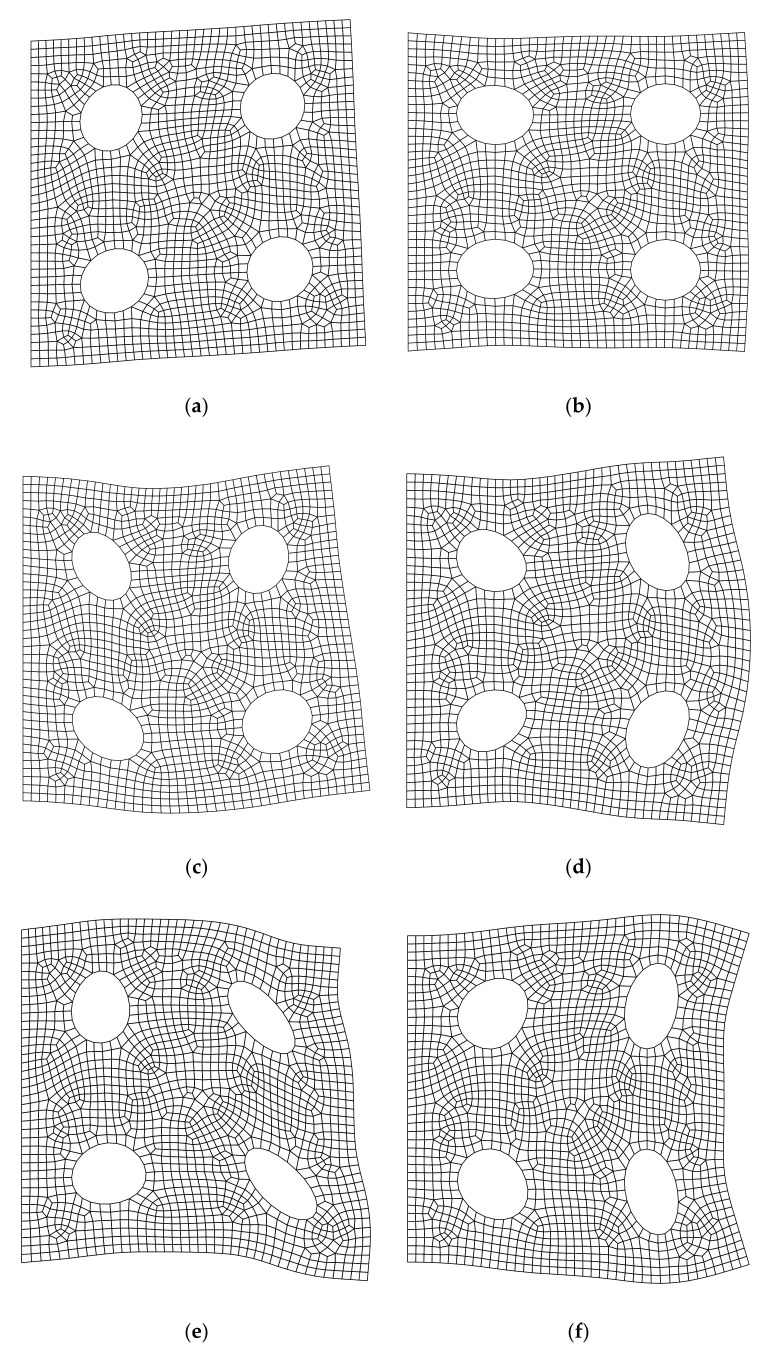
First 6 mode shapes of the plate with four holes using FE-RPIM QUAD4 element (CMM): (**a**) Mode 1; (**b**) Mode 2; (**c**) Mode 3; (**d**) Mode 4; (**e**) Mode 5; (**f**) Mode 6.

The first six mode shapes obtained through the FE-RPIM QUAD4 element under CMM are presented in Figure 19. Mode shapes through CMM agree well with those through DLMM in the context of FE-RPIM QUAD4 element.

### 4.6. A Cantilever Beam under Harmonic Load

In the context of FE-RPIM QUAD4 element, accuracy for natural frequencies obtained through the DLMM is as good as or even better than those through the CMM. This conclusion was also hold FEM [61,63]. However, the CMM is considered to be a better choice to calculate deformation and mode shapes [61,63]. In this section, following the same purpose as in [61], an example is employed to show that the FE-RPIM QUAD4 element is able to obtain satisfactory results by employing DLMM even for the scheme of implicit time integration.

**Figure 20:**
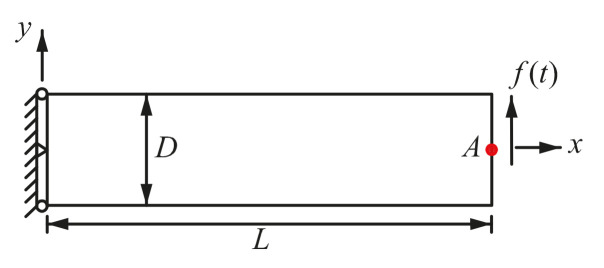
A 2D cantilever beam subjected to a harmonic loading on the right end.

A cantilever beam (Figure 20) under harmonic load ($f\left(t\right)$) on the right end is considered. The parameters used are  ρ = 1 kg/m^3^, *D* = 1 m, *L* = 4 m, *v* = 0.3, *E* = 1 Pa, $f\left(t\right)=\sin\omega_{f}t$, $\omega_{f}=0.04\;rad/s$, $\beta_{1}=0.005$ and $\beta_{2}=0.272$.

**Figure 21:**
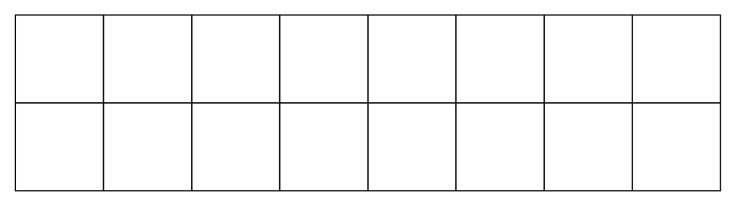
Discretized model of the 2D cantilever beam subjected to a harmonic loading.

In the computation, scheme of implicit time integration with γ=1 and β=0.5 is used. Time step size Δt=1.57s is adopted. Shown in Figure 21 is the discretized model used by the FE-RPIM QUAD4 element. Apart from the FE-RPIM QUAD4 element, this dynamic problem is also investigated using QUAD4 element (27 nodes, 16 elements), TRIG3 element (27 nodes, 32 elements) and QUAD8 element (69 nodes, 16 elements). In order to compute a reference solution, the QUAD4 element is adopted with a very dense discretized model (6601 nodes, 6400 elements).

**Figure 22:**
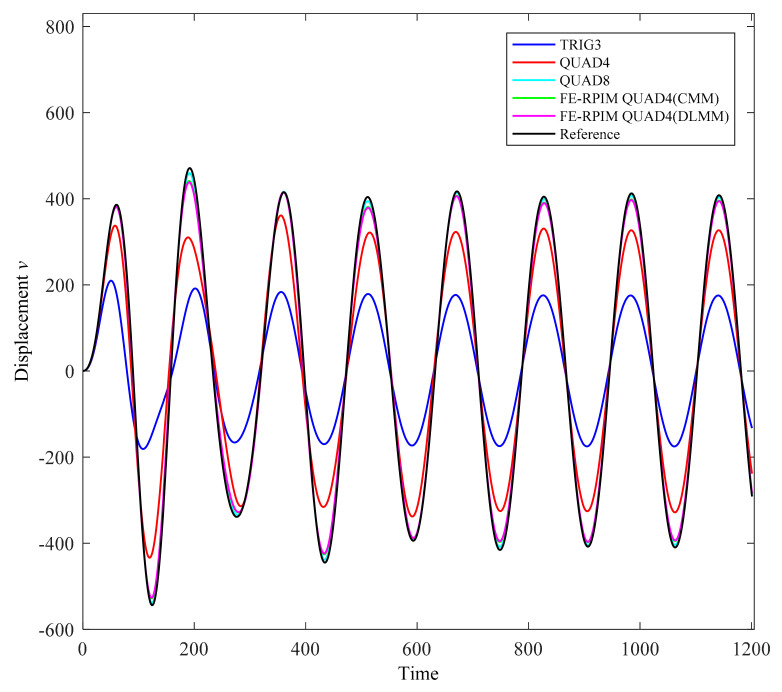
Transient responses of a cantilever beam subjected to a harmonic loading.

As can be seen from Figure 22, if CMM scheme is employed, FE-RPIM QUAD4 element’s accuracy is higher than QUAD4 and TRIG3 elements. More importantly, the result of FE-RPIM QUAD4 (DLMM) almost coincides with FE-RPIM QUAD4 (CMM), which means DLMM can supersede the CMM in the context of the FE-RPIM QUAD4 element.

## 5. Conclusions

FE-RPIM QUAD4 element is further extended for problems of structural dynamics. Some important conclusions, which can be drawn from this work, are as follows:

(1) Based on 4-node quadrilateral mesh, FE-RPIM QUAD4 element’s accuracy is much higher than QUAD4 and TRIG3 elements (Table 2).
(2) Although FE-RPIM QUAD4 element’s accuracy is slightly inferior to QUAD8 element, QUAD8 element requires more nodes than FE-RPIM QUAD4 element to discretize the problem domain. In addition, FE-RPIM QUAD4 element can achieve results closing to the reference solution, even for coarse mesh (Figure 22).
(3) For distorted meshes, FE-RPIM QUAD4 element’s accuracy is always much higher than QUAD4 and TRIG3 elements. Moreover, FE-RPIM QUAD4 element is immune to mesh distortion, but TRIG3, QUAD4 and QUAD8 elements give very bad results as the mesh quality deteriorates (Figure 14).
(4) In the tests associated to the analysis of free vibration, the result based on the DLMM are very close to those based on the CMM in the context of FE-RPIM QUAD4 element. In the test on forced vibration analysis, the result from the DLMM also agrees well with that from the CMM, which means DLMM can supersede the CMM in the context of the FE-RPIM QUAD4 element even for the scheme of implicit time integration.

## Figures and Tables

**Table 1:** Comparisons of computed frequencies (Hz) for the slender rod with regular.

Mesh	Mode	TRIG3	QUAD4	QUAD8	FE-RPIM QUAD4 (CMM)	FE-RPIM QUAD4 (DLMM)	Analytical Solution [62]
Mesh A (100 × 1)	1	25.820968	25.820965	25.819889	25.820844	25.820870	25.819889
	2	51.648164	51.645511	51.639778	51.636459	51.647617	51.639778
	3	77.487948	77.485357	77.459667	77.333589	77.486075	77.459667
	4	103.346621	103.353319	103.279556	103.770772	103.341991	103.279556
	5	129.231393	129.235285	129.099445	129.144371	129.220982	129.099445
	6	155.144150	155.090370	154.919334	154.271801	155.128495	154.919334
	7	181.097287	181.093361	180.739223	179.861768	181.069767	180.739223
	8	207.100128	207.044472	206.559112	207.167158	207.049784	206.559112
	9	233.139899	233.163742	232.379001	231.620531	233.073245	232.379001
	10	259.236693	259.205890	258.198890	257.916223	259.144523	258.198890
Mesh B (200 × 2)	**Mode**	**TRIG3**	**QUAD4**	**QUAD8**	**FE-RPIM QUAD4 (CMM)**	**FE-RPIM QUAD4 (DLMM)**	**Analytical Solution [62]**
	1	25.820157	25.820255	25.819884	25.819736	25.819876	25.819889
	2	51.641819	51.643160	51.639488	51.642793	51.639674	51.639778
	3	77.466851	77.468030	77.460498	77.428906	77.459316	77.459667
	4	103.296728	103.295143	103.276931	103.223462	103.278723	103.279556
	5	129.132375	129.129519	129.098294	129.103269	129.097812	129.099445
	6	154.976563	154.971476	154.916664	154.910322	154.916499	154.919334
	7	180.830533	180.849114	180.739424	180.695709	180.734699	180.739223
	8	206.695973	206.729136	206.571040	206.625255	206.552319	206.559112
	9	232.574473	232.597669	232.373245	232.226958	232.369264	232.379001
	10	258.459661	258.424349	258.192677	258.548565	258.185433	258.198890
Mesh C (400 × 4)	**Mode**	**TRIG3**	**QUAD4**	**QUAD8**	**FE-RPIM QUAD4 (CMM)**	**FE-RPIM QUAD4 (DLMM)**	**Analytical Solution [62]**
	1	25.819955	25.819955	25.819872	25.819892	25.819889	25.819889
	2	51.640264	51.640326	51.639657	51.639523	51.639777	51.639778
	3	77.461418	77.461344	77.455360	77.458972	77.459663	77.459667
	4	103.283785	103.284022	103.280289	103.281014	103.279545	103.279556
	5	129.107774	129.107848	129.098050	129.100114	129.099422	129.099445
	6	154.933575	154.933779	154.917190	154.920049	154.919292	154.919334
	7	180.762060	180.761938	180.731242	180.738237	180.739152	180.739223
	8	206.593046	206.592175	206.540550	206.564266	206.558998	206.559112
	9	232.427179	232.427476	232.386967	232.371678	232.378826	232.379001
	10	258.265837	258.264934	258.234066	258.188904	258.198630	258.198890

**Table 2:** Comparisons of computed frequencies (Hz) for the annulus.

Mesh	Mode	TRIG3	QUAD4	QUAD8	FE-RPIM QUAD4 (CMM)	FE-RPIM QUAD4 (DLMM)	Reference Solution [61]
Mesh A	1	1069.0	764.6	331.6	465.7	459.3	307.3
	2	1069.0	765.7	331.6	465.8	459.3	307.3
	3	1973.0	1917.4	945.3	1683.8	1623.6	838.5
	4	2759.1	2346.5	945.3	1686.7	1623.6	838.5
	5	2760.6	2350.0	1823.4	1938.7	1937.9	1535.4
	6	2779.7	2775.5	1823.9	2665.5	2714.8	1535.4
Mesh B	**Mode**	**TRIG3**	**QUAD4**	**QUAD8**	**FE-RPIM QUAD4 (CMM)**	**FE-RPIM QUAD4 (DLMM)**	**Reference Solution [61]**
	1	601.2	430.5	310.7	318.9	317.8	307.3
	2	601.2	430.5	310.7	318.9	317.8	307.3
	3	1622.2	1221.1	851.1	895.7	890.0	838.5
	4	1622.2	1221.3	851.1	895.8	890.0	838.5
	5	1869.2	1855.6	1566.6	1689.0	1665.2	1535.4
	6	2619.8	2351.4	1567.6	1691.0	1665.2	1535.4
Mesh C	**Mode**	**TRIG3**	**QUAD4**	**QUAD8**	**FE-RPIM QUAD4 (CMM)**	**FE-RPIM QUAD4 (DLMM)**	**Reference Solution [61]**
	1	402.7	340.1	308.0	308.0	307.8	307.3
	2	402.7	340.1	308.0	308.0	307.8	307.3
	3	1098.2	938.0	840.6	841.7	841.2	838.5
	4	1098.4	938.0	840.6	841.7	841.2	838.5
	5	1843.9	1742.3	1539.8	1544.0	1542.4	1535.4
	6	2013.4	1742.4	1539.9	1544.0	1542.4	1535.4
Mesh D	**Mode**	**TRIG3**	**QUAD4**	**QUAD8**	**FE-RPIM QUAD4 (CMM)**	**FE-RPIM QUAD4 (DLMM)**	**Reference Solution [61]**
	1	333.8	315.7	307.4	307.4	307.4	307.3
	2	333.8	315.7	307.4	307.4	307.4	307.3
	3	911.4	863.7	839.0	839.0	839.0	838.5
	4	911.4	863.8	839.0	839.0	839.0	838.5
	5	1670.8	1587.3	1536.3	1536.5	1536.6	1535.4
	6	1670.8	1587.6	1536.3	1536.5	1536.6	1535.4

**Table 3:** Computed natural frequencies (Hz) of the first mode for the mesh distortion sensitivity test.

2*d*/*D*	TRIG3 (CMM)	QUAD4 (CMM)	QUAD8 (CMM)	FE-RPIM QUAD4 (CMM)	FE-RPIM QUAD4 (DLMM)	Reference Solution [61]
0.000	4140.56	2623.12	868.78	1024.59	984.12	822.13
0.025	4296.89	2709.93	871.92	1028.00	986.58	822.13
0.050	4444.46	2888.54	880.81	1033.54	989.62	822.13
0.075	4556.81	3052.25	894.10	1037.37	989.81	822.13
0.100	4642.17	3168.80	910.07	1039.62	987.54	822.13
0.150	4772.43	3294.48	947.03	1041.76	979.18	822.13
0.200	4880.56	3350.17	999.17	1042.67	969.09	822.13
0.250	4979.99	3382.91	1085.88	1043.14	958.78	822.13
0.300	5074.54	3412.02	1219.18	1043.44	948.37	822.13
0.400	5255.27	3484.76	1593.73	1043.84	925.26	822.13
0.500	5428.50	3586.06	1988.16	1044.17	894.74	822.13
0.600	5596.39	3714.14	2309.06	1044.52	853.35	822.13
0.700	5759.40	3867.25	2551.71	1044.91	802.31	822.13
0.800	5919.14	4040.12	2741.70	1045.38	747.99	822.13
0.900	6073.71	4229.27	2904.91	1045.94	698.62	822.13

**Table 4:** Comparisons of computed frequencies (Hz) for the plate with four holes.

Mesh	Mode	TRIG3	QUAD4	FE-RPIM QUAD4 (CMM)	FE-RPIM QUAD4 (DLMM)	Reference Solution
Mesh A	1	49.21	48.60	48.24	48.26	47.93
	2	118.15	117.29	116.73	116.72	116.25
	3	129.69	128.04	126.93	126.96	126.13
	4	209.36	206.57	204.58	204.76	203.25
	5	214.32	210.56	207.54	207.42	205.34
	6	235.48	232.65	230.54	230.80	229.13
Mesh B	**Mode**	**TRIG3**	**QUAD4**	**FE-RPIM QUAD4 (CMM)**	**FE-RPIM QUAD4 (DLMM)**	**Reference Solution**
	1	48.85	48.39	48.11	48.12	47.93
	2	117.70	116.97	116.55	116.54	116.25
	3	128.71	127.38	126.61	126.63	126.13
	4	207.63	205.38	204.02	204.10	203.25
	5	211.84	208.73	206.59	206.53	205.34
	6	233.86	231.49	229.97	230.00	229.13
Mesh C	**Mode**	**TRIG3**	**QUAD4**	**FE-RPIM QUAD4 (CMM)**	**FE-RPIM QUAD4 (DLMM)**	**Reference Solution**
	1	48.65	48.26	48.05	48.05	47.93
	2	117.37	116.79	116.46	116.46	116.25
	3	128.19	127.06	126.46	126.45	126.13
	4	206.78	204.86	203.79	203.76	203.25
	5	210.77	207.83	206.20	206.12	205.34
	6	233.10	231.02	229.73	229.83	229.13
Mesh D	**Mode**	**TRIG3**	**QUAD4**	**FE-RPIM QUAD4 (CMM)**	**FE-RPIM QUAD4 (DLMM)**	**Reference Solution**
	1	48.40	48.13	47.99	47.99	47.93
	2	116.92	116.55	116.34	116.34	116.25
	3	127.41	126.65	126.27	126.27	126.13
	4	205.48	204.17	203.49	203.46	203.25
	5	208.73	206.80	205.72	205.68	205.34
	6	231.49	230.13	229.39	229.44	229.13

## Data Availability

The data presented in this study are available on request from the corresponding author.
